# Iatrogenic Sinistral Hypertension Complicating Screening Colonoscopy

**DOI:** 10.1155/2013/695318

**Published:** 2013-08-19

**Authors:** Oliver J. Ziff, A. M. James Shapiro

**Affiliations:** Department of Surgery, University of Alberta, 2000 College Plaza, 8215 112th Street, Edmonton, AB, Canada T6G 2C8

## Abstract

Colonoscopy is widely accepted as the gold-standard screening technique for detecting malignancies in the distal gastrointestinal tract in patients with symptoms suggestive of colon cancer. However, this procedure is not without risk, including colonic perforation. We report a patient who was managed conservatively after colonoscopy induced perforation. Eighteen months after appearing to make a full recovery, he presented with an upper gastrointestinal bleed. Oesophago-gastro-duodenoscopy (OGD) revealed large gastric fundal varices and computed tomography (CT) revealed splenic vein thrombosis. The ensuing left-sided (sinistral) hypertension explains the development of the fundal varices in the presence of normal liver function. At surgery, a persistent abscess cavity was identified and cultures from this site grew *Streptococcus anginosus*. Curative splenectomy was performed and the patient made a full recovery. We advocate more prompt operative intervention in selected cases of iatrogenic colonic perforation with primary repair to prevent late complications.

## 1. Introduction

**Figure 1:**
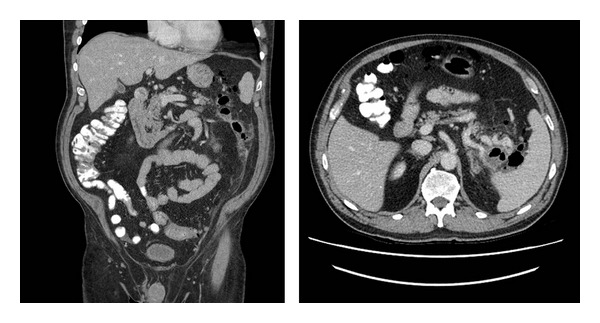
Perforation showing free air in the peritoneum in the area of splenic flexure.

A 69-year-old male presented with a one-week history of postural hypotension and melena. On physical examination, he appeared anemic, and a digital rectal examination confirmed melena. The examination was otherwise unremarkable, and there was no peripheral stigmata of chronic liver disease. Eighteen months previously he underwent a screening colonoscopy where diverticulosis and 8 polyps were snared, removed, and retrieved, varying in size between 5 and 25 mm. Histopathology confirmed benign tubular, tubulovillous adenomas, and adenomatous polyps. Following that procedure he developed a localized perforation in the area of the splenic flexure, with free air and abscess (Figure 1). This was treated conservatively, and he subsequently appeared to make a full recovery. 

**Figure 2:**
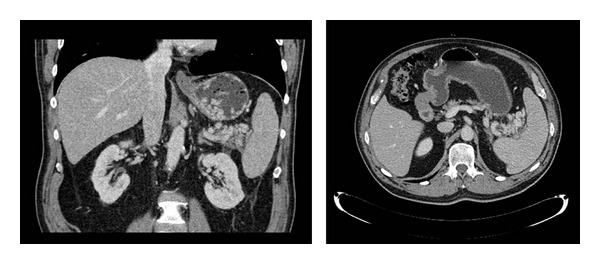
Cluster of varices in the left upper quadrant medial to the splenic hilum with multiple gastric varices within the gastric lumen.

Blood tests confirmed anemia (Hb 121 g/L, PLT 186 × 10^9^ g/L), with normal liver function and prothrombin time. Assessment of his upper gastrointestinal bleed by oesophagogastroduodenoscopy (OGD) revealed large gastric fundal varices. Subsequent computed tomography (CT) of the abdomen with triple-phase contrast demonstrated a cluster of varices in the left upper quadrant (Figure 2). The splenic vein was thrombosed over a narrow segment between the tail of the pancreas and spleen. The ensuing left-sided (sinistral) portal hypertension explains the development of gastric varices in the presence of normal liver function. Since these varices were actively bleeding, curative splenectomy was deemed the most appropriate management after administration of presplenectomy vaccinations [1].

At open surgery, extensive vascular adhesions were observed around the splenic flexure of the colon, splenic hilum, and pancreatic tail. Dissection of the transverse mesocolon revealed a persistent abscess cavity that had failed to resolve over 18 months, resulting from the previous colonoscopic perforation, and cultures from this site grew *Streptococcus anginosus*. The abscess measured 3 cm × 2 cm, and due to the thrombosis and the abscess location, it was not amenable to percutaneous drainage. Following ligation of the splenic artery in the lesser sac, the sinistral varices decompressed, allowing safe splenectomy. Histopathology showed splenic fibrosis, inflammation, and no malignancy. He made an uncomplicated recovery from surgery with no further gastric bleeding with 10 months of followup.

## 2. Discussion

Patients with sinistral portal hypertension frequently form varices most commonly in the fundus of the stomach. This location is explained by the venous drainage of the spleen via the short gastric veins. In contrast, gastric and oesophageal varices are more commonly precipitated by hepatic sinusoidal hypertension, but these patients usually exhibit other stigma of chronic liver disease [2] which are absent in presinusoidal portal hypertension. We herein describe a case of sinistral hypertension with recurrent upper gastrointestinal bleeding resulting from splenic venous thrombosis, as a late complication of splenic flexure colonic perforation. This complication has not been described previously to our knowledge.

Colonoscopy is an invaluable tool in assessing diseases of the rectum and colon; however, it is in an invasive procedure and not without complications. Perforation and bleeding occur in 0.12% of patients undergoing colonoscopy, and peritoneal abscesses have been reported [3]. Such patients may benefit from prompt laparotomy with primary repair or limited resection with anastomosis to minimise morbidity and mortality [4]. Conservative management may on occasion be entirely appropriate, but on rare occasion this course may lead to added risk of further complications, as illustrated by the case described.

Colonic perforation with development of a peritoneal abscess leading to splenic vein thrombosis is clearly a very rare iatrogenic cause of sinistral portal hypertension and variceal bleeding. More common causes of splenic vein thrombosis include pancreatic tumours, pancreatitis, pancreatic pseudocysts, splenic vein stenosis, and polycystic disease of the liver and pancreas [5–7]. If the common risk factors for upper GI haemorrhage are absent, including NSAID use and cirrhosis, then OGD and imaging of the abdomen are required to rule out splenic vein thrombosis. 

In conclusion, conservative management of an iatrogenic colonoscopic perforation at the splenic flexure led to late presentation of life-threatening sinistral portal hypertension with gastric fundal varices secondary to splenic vein thrombosis and sinistral portal hypertension. In cases of active bleeding, splenectomy is indicated and can be curative [1]. We advocate more prompt operative intervention in cases of splenic flexure colonic perforation, where the perforation is localized to the lesser sac. Primary repair following iatrogenic gastrointestinal perforation may have prevented a late complication that was more challenging to manage from a surgical perspective.
